# The drainage tube into the dural-outer membrane space during burr hole evacuation for chronic subdural hematoma: Novel case report of 2 patients

**DOI:** 10.1097/MD.0000000000043330

**Published:** 2025-07-11

**Authors:** Cheng Wang, Kun Zhou, Junquan Chen, Jiacai Qian, Chengming Zhou, Xiaopeng Deng, Xiaolin Du

**Affiliations:** a Department of Neurosurgery, The Jinyang Hospital Affiliated to Guizhou Medical University (The Second People’s Hospital of Guiyang), Guiyang, Guizhou Province, China.

**Keywords:** burr hole, case report, chronic subdural hematoma, drain misplacement, outer membrane, subdural drainage

## Abstract

**Rationale:**

During burr hole evacuation for chronic subdural hematoma (cSDH), the drainage catheter may inadvertently enter the potential space between the dura mater and the hematoma’s outer membrane (dural-outer membrane (DOM) space), an unrecognized complication. This misplacement can lead to inadequate drainage, delayed recovery, and recurrence. This report describes 2 cases illustrating this phenomenon and aims to raise awareness and propose preventative strategies.

**Patient concerns:**

Two male patients (mean age: 77 years) presented with symptoms including limb weakness, headache, or dizziness.

**Diagnoses:**

Computed tomography scans revealed that case 1 had bilateral frontotemporoparietal high-density subdural hematomas, while case 2 exhibited a trabecular-septated subdural hematoma in the right frontotemporal region. The preoperative diagnosis was chronic subdural hematoma (possibly with intracranial hypertension).

**Interventions:**

Preoperative evaluation localized the hematoma, and burr hole evacuation was performed at the site of maximum thickness. Postoperatively, minimal or no hematoma drainage was observed in both patients. One patient experienced hematoma recurrence, while the other had prolonged hospitalization and delayed symptom resolution.

**Outcomes:**

Retrospective analysis of the surgical procedures, imaging studies, and clinical outcomes confirmed that the drainage catheter had entered the DOM space in both cases. This misplacement was identified as the cause of the poor drainage and adverse outcomes.

**Lessons:**

Recognizing the risk of catheter misplacement into the DOM space during burr-hole evacuation is essential, as it can significantly impair therapeutic efficacy. To our knowledge, this is the first report documenting catheter entry into the DOM space. The clinical insights and preventative measures proposed here may help avoid this complication and improve surgical outcomes in future cases.

## 1. Introduction

With the global rise in population aging, the incidence of chronic subdural hematoma (cSDH) is steadily increasing.^[1]^ Although there is no universally accepted surgical standard, most neurosurgeons choose burr hole evacuation; adjunctive catheter-based continuous drainage reduces recurrence rates.^[2,3]^ The outer membrane of cSDH comprises several components, including fragile neovasculature and local fibrinolytic agents,^[4]^ and is considered a key contributor to recurrence.^[5,6]^ Usually, the adhesion between the dura mater and the outer membrane is weak, increasing the risk of the catheter entering the dural-outer membrane (DOM) space. This displacement can delay recovery and potentially contribute to hematoma recurrence.

## 2. Case presentation

The work was approved by the Ethics Committee of The Second People’s Hospital of Guiyang (JYYY-2025-WZ-16). The patient has given his written informed consent for this study and the publication of his related data.

### 2.1. Case 1

**Figure 1.**
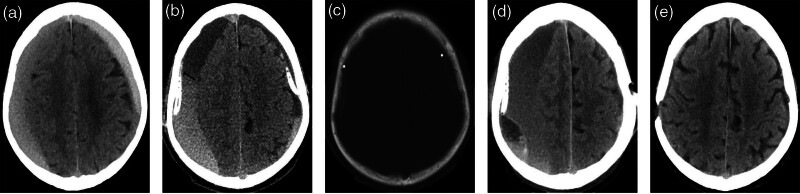
Axial CT scans of case 1. (A) Preoperative image; (B) a linear high-density lesion (black arrow) at the anterior edge of the drainage tube suggests outer membrane detachment and bleeding at 12 h postoperatively; (C) a bone window showing the drainage tube closely adhered to the skull; (D) increased right-sided subdural hematoma at 56 h postoperatively; (E) resolution of cSDH and return to neurologic baseline at 69 d postoperatively. cSDH = chronic subdural hematoma, CT = computed tomography.

An 80-year-old man presented with bilateral lower limb weakness and a history of traumatic brain injury approximately 2 months prior. Cranial computed tomography (CT) revealed bilateral cSDH (Fig. 1A). The patient underwent bilateral burr hole evacuation. Surgical findings revealed a tense dura mater, and the hematoma’s outer membrane was visualized on durotomy. Upon incision of the outer membrane, a pressurized, motor oil-colored subdural clot was released, followed by copious irrigation with normal saline (NS) and a subdural drainage catheter placement. Twelve hours postoperatively, CT showed a residual right hematoma measuring 22.3 mm (Fig. 1B), and the catheter appeared tightly affixed to the skull (Fig. 1C). The drainage volume remained low (3–5 mL/d). At 56 hours postoperatively, the patient’s level of consciousness deteriorated to E3M4V6 on the Glasgow Coma Scale. Repeat CT demonstrated hematoma progression on the right (Fig. 1D), necessitating reoperation. A new catheter was correctly positioned in the hematoma cavity. The cranial CT at 12 hours postoperation confirmed marked hematoma reduction. At the 69-day follow-up, the patient had returned to his neurological baseline, with near-complete hematoma resolution on CT (Fig. 1E).

### 2.2. Case 2

**Figure 2.**
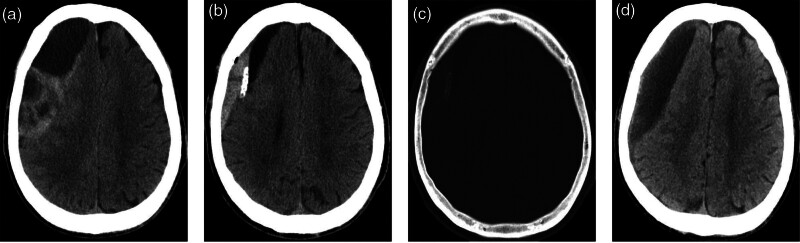
Axial CT scans of case 2. (A) Preoperative image; (B) acute right-sided subdural hemorrhage at 19 h postoperatively; (C) hemorrhagic lesion encasing the drainage tube; (D) increased subdural effusion at 30 days postoperatively. CT = computed tomography.

The patient was a 74-year-old man with a history of head trauma over 2 months prior. He presented with headache, dizziness, and left limb weakness. Cranial CT revealed a right cSDH (Fig. 2A). Intraoperatively, the outer membrane of the hematoma appeared thickened. A large volume of NS was used for irrigation until the effluent was clear, and a subdural catheter was placed. Nineteen hours postoperatively, a follow-up cranial CT revealed acute hemorrhage (Fig. 2B), with the hematoma encasing the catheter (Fig. 2C). The drainage tube had dissected the DOM interface, causing bleeding from the outer membrane of the cSDH.^[7]^ Postoperative drainage was minimal, ranging from 5–10 mL/d. At 30-day follow-up, although the patient’s symptoms had gradually improved, CT imaging demonstrated increased subdural effusion (Fig. 2D).

## 3. Discussion

The pathophysiology of cSDH remains unclear. Research on the outer and inner membranes of cSDH is ongoing. The outer membrane comprises fibroblasts and collagen fibers. Typically, the adhesion between the dura mater and the outer membrane is loose, making it susceptible to detachment and hemorrhage.^[7]^ Inadequate catheter placement may lead to insufficient drainage and poor surgical outcomes. To our knowledge, this is the first report documenting catheter insertion into the DOM space. We have summarized the potential causes and preventive strategies to inform neurosurgical practice.

The catheter migrates into the DOM space primarily due to the presence of the outer membrane, which is a necessary condition. Typically, the adhesion between the outer membrane and the dura is loose, allowing the catheter to dissect into the DOM space.

Clinically, catheter misplacement into the DOM space is suggested when intraoperative hematoma liquefaction appears adequate, but postoperative drainage is minimal or absent. Negative pressure suction fails to extract fluid, while NS can be injected easily, indicating the catheter is patent but misplaced. Postoperative CT scans usually show the catheter closely adherent to the dura, running along the outer membrane surface, which is prone to bleeding.

Thirdly, avoiding catheter placement into the DOM space is crucial. Enlarging the burr hole facilitates smoother entry of the catheter into the hematoma cavity rather than the DOM space. If hematoma fluid is not observed exiting the catheter during flushing using the siphon method,^[8]^ DOM space misplacement and poor drainage could be indicated, warranting catheter repositioning. Flushing in multiple directions before placing the catheter is advisable to minimize residual hematoma. Moreover, subgaleal drainage may be considered, as it does not increase the recurrence rate.^[9]^

In cases of catheter misplacement, appropriate management is essential. For patients with significant residual hematoma but no symptoms, oral administration of atorvastatin may aid in hematoma absorption,^[10]^ and dexamethasone can be added to enhance therapeutic effects.^[11]^ However, if symptoms persist or worsen, a second burr hole procedure with drain repositioning is recommended. Burr hole evacuation should be prioritized over craniotomy in recurrent cases to maintain surgical simplicity.

## 4. Limitations

First, most cSDHs have a thin outer membrane, making it challenging to identify catheter misplacement into the DOM space via postoperative MRI. However, diffusion-weighted imaging may offer diagnostic value.^[12]^ Second, the most definitive method to confirm the position of the catheter is to reserve it for inspection during craniotomy. However, this method increases infection risk due to prolonged catheter retention. In most cases, a second burr hole procedure with catheter adjustment effectively resolves the hematoma, eliminating the need for craniotomy.

## 5. Conclusion

Catheter entry into the DOM space can lead to inadequate drainage, residual hematoma, delayed recovery, and potential recurrence. This complication may be preventable by applying the surgical insights and strategies outlined above.

## Acknowledgments

We thank Bullet Edits Limited for providing the manuscript’s linguistic editing and proofreading services and Guangming Du, father of the corresponding author, for his support.

## Author contributions

**Conceptualization:** Cheng Wang, Xiaolin Du.

**Data curation:** Xiaolin Du.

**Formal analysis:** Cheng Wang, Xiaolin Du.

**Funding acquisition:** Xiaolin Du.

**Resources:** Cheng Wang.

**Supervision:** Cheng Wang, Kun Zhou.

**Writing – original draft:** Cheng Wang, Kun Zhou, Junquan Chen, Jiacai Qian, Chengming Zhou, Xiaopeng Deng, Xiaolin Du.

**Writing – review & editing:** Cheng Wang, Xiaolin Du.
